# History of Gap Junction Architecture and Potential Role of Calmodulin in Channel Arrays

**DOI:** 10.3390/ijms262311337

**Published:** 2025-11-24

**Authors:** Camillo Peracchia

**Affiliations:** Department of Pharmacology and Physiology, School of Medicine and Dentistry, University of Rochester, Rochester, NY 14642-8711, USA; camillo_peracchia@urmc.rochester.edu

**Keywords:** gap junctions, connexin, innexin, calcium, calmodulin, cell communication, cell-cell channels, cell coupling, cell uncoupling, channel gating

## Abstract

This review article focuses first on the historical development of present understanding of gap junction channel architecture, one of its goals being to enlighten younger generations of scientists about the early steps of this field that begun over half a century ago. Early findings on gap junction architecture are reviewed as follows. The channels cross the membrane and project from the membrane surfaces; they are made of six subunits (hexamers) and show dimples on both ends, which represent inner and outer openings of the channel. Images of the central dimples on both channel ends (channel pores) seen in freeze-fracture replicas correspond to the electron-opaque spots visible in negatively stained sections and in isolated junctions. The channels are linked to each other extracellularly. Calmodulin (CaM) is a major accessory protein of gap junctions that is involved in channel gating and gap junction formation and is also likely to play a key role in determining different patterns of channel aggregation.

## 1. Introduction

This review addresses, first, the historical development of present knowledge of gap junction architecture by briefly describing the major characteristics of early models of gap junction channel arrays. Next, it discusses evidence for different channel arrays, the potential role of calmodulin (CaM) in channel aggregation, and the relationship between channel arrays and channel permeability.

Most scientists who study or have studied direct intercellular communication via gap junctions are aware that the channels cluster at cell–cell contact in different types of arrays, varying from disordered channel aggregations to more orderly, and mostly hexagonal, arrays. Only a few studies, however, have considered the possibility that different patterns of channel aggregation reflect different states of channel permeability. In our studies on gap junction ultrastructure, we have often addressed this issue and over the years we have provided experimental data and hypotheses on the functional meaning of channel arrays. Note, however, that this review article does not cover the recent data on a single channel’s molecular structure, which have been recently revealed in detail by cryo-electron microscopy. Briefly, the cryoEM maps demonstrate the presence of lipid densities in the pore, within transmembrane α-helices and in protomers, whose functions are still unclear. Both cytoplasmic loop and C-terminal sequences are not seen probably because of their flexibility. The cryoEM structure of the hexameric connexon in lipid bilayer shows a 4-helix transmembrane arrangement very similar to that of dodecameric channels. While resolution of the transmembrane α-helices is excellent, the resolution of the extracellular loops is poor. The flexibility of the extracellular loops facilitates the intercellular docking. For a review on single channel structure, see refs. [1,2].

It should be mentioned to the young readers who may not be aware of early steps in our understanding of gap junction structure that the limitations of early techniques prevented, for many years, a full understanding of the structure of cell–cell channels. Presently, modern techniques such as protein crystallization and cryo-electromicroscopy have greatly contributed to our detailed knowledge of channel molecular structure. However, as mentioned previously, this review is focused on gap junction architecture rather than channel molecular structure. 

## 2. Early Evidence for Direct Cell-to-Cell Communication

The discovery of electrical cell–cell communication, first reported in septate giant axons of invertebrates [3,4], surprised physiologists of the early 20th century, as it was clear that neighboring axonal segments were not linked by syncytia-like cytoplasmic bridges but were, in fact, individual cells limited by obvious barriers [5,6,7]. How could electrical current spread rapidly across these barriers? This question started being answered following the invention of the electron microscope. Several decades later, evidence for the existence of membrane channels that mediate direct cell-to-cell communication was also demonstrated in (electrically) non-excitable cells [8,9,10,11].

## 3. Early Ultrastructural Images of Gap Junctions in Excitable Cells of Invertebrates

The earliest electron micrographs of junctions linking neighboring segments of crayfish giant axons were published in the mid-1950s by J. David Robertson [12,13]; in his words, “… *the motor fiber axon-Schwann membrane, which envelopes the synaptic processes of motor axoplasm fuses with the median giant axon-Schwann membrane to form a single membrane of structure apparently identical with that of either of the fusing membranes … to produce the axon-axon synaptic membrane*” [13]. Soon after Robertson’s publication, similar images were reported by Marietta Issidorides [14] in axons and dendrites of earthworm neuropiles; in her words, “… *All recent investigations of the synapse with the electron microscope … agree to the fact that axo-axonal, axo-dendritic and axosomatic synapses are characterized by the close apposition or contact of two plasma membranes contributed by each member of a pair*”. However, Issidorides did not notice that the two plasma membranes were linked to each other at the junction; instead, she wrote: “… *In the giant axons, however, the synapses are characterized by the interposition of several myelin lamellae between pre- and postsynaptic axoplasms. Since these macrosynapses have been found to be very little delaying … compared to other synapses the large amount of myelin present at these sites not only lacks an insulating function, as it is generally believed for this substance, but seems to be expedient in some way to the passage of the nerve impulse*” [14]. Nonetheless, Issidorides deserves credit for suggesting that these junctions are likely to be involved in impulse propagation.

In early studies, the profiles of junctional membranes were described as individual electron-dense lines, because early electron micrographs did not have sufficient resolution to view the membrane structure in detail. The actual trilaminar profile of the membrane, composed of a 25 Å wide electron-transparent space sandwiched between two 30 Å thick electron-dense lines, was first reported in 1957 by J. D. Robertson, who named this structure “*unit membrane*” [15,16]. Three years later, A. J. Darin De Lorenzo also noticed the trilaminar membrane profile in junctions linking crayfish lateral giant axons to motor axons [17]; in his words, “… *The appositional membranes are separated by a small space approximately 50–75 Å wide … Each of the appositional membranes is composed of a structural unit consisting of two electron dense lines, about 25 Å thick, separated by a light zone about 30 Å wide*”. A year later, similar images were also published by Kiyoshi Hama [18].

A remarkable study published by Hama on junctions between earthworm giant axons [19] was first to notice the presence of a gap between the junctional membranes; in his words, “… *The two plasma membranes are separated from each other by a gap of about 65 Å (40–85 Å). Together they form a pair of membranes 200 Å (150–250 Å) wide over-all. … In some places characteristic vesicles are associated with the septal membranes… These vesicles are about 160 Å in diameter, are remarkably uniform, and are arranged in an orderly way on the cytoplasmic surfaces of each of the two membranes composing the septum. The vesicles are oriented and regularly spaced, and often face a similar fellow vesicle in a symmetrical apposing position on the adjacent membrane of the neighboring neural unit*”. Hama reported that the vesicles are similar to synaptic vesicles; however, this is impossible because biological membranes do not bend sharply enough to create vesicles smaller that the ~400 Å synaptic vesicles [20]. In retrospect, based on our study on gap junctions between crayfish axons [21], what Hama described [19] was probably the cytoplasmic protrusions of gap junction channels and accessory molecules (see in the following).

## 4. Gap Junctions in Vertebrates—Early Images

### 4.1. Pentalaminar Junction Profile

The first micrographs of gap junctions in vertebrates were published in the late 1950s by Fritiof S. Sjöstrand and coworkers [22], who named the junctions “*longitudinal connecting surfaces”*. In their words, “*In the mouse cardiac muscle, the two plasma membranes in close contact give rise to a layered structure with five layers, three opaque and two less opaque interposed layers. The middle opaque layer is thinner than the two others, which measure 40 Å in thickness. It divides the 90 Å wide interspace between the other two opaque layers into two halves*”. This report, however, did not entertain the possibility that pentalaminar junctions are involved in electrical cell–cell coupling, as it focused primarily on data suggesting the independent, rather than interactive, nature of cardiac cells.

Similar images, published by Henry E. Karrer in cervical epithelium [23] and venous smooth muscle [24], were called “*quintuple-layered interconnections*”. The images were of higher resolution, and these junctions were thought to be involved in cell–cell communication between smooth muscle cells; in his words, “*… they provide for the closest possible contact between separate muscle cells. It therefore appears permissible to speculate that the quintuple-layered interconnections might be of significance in the cell-to-cell conduction of excitation in heart muscle*” [24]. A similar pentalaminar junction, named “*nexus*”, was described by M.M. Dewey and L. Barr in smooth muscle cells of dog’s intestine [25]. These authors also recognized that these junctions mediate cell-to-cell electrical coupling; in their words, “… *In smooth muscle fixed in permanganate, a nexus comprises three dark lines of uniform thickness separated by two lighter regions… Wherever a nexus exists, the resulting direct fusion of the outer layers of the cell membranes without intervening extracellular space allows electrotonic spread of current from one cell membrane to another in a way similar to its spread along an axon. This is probably the mechanism for propagation in smooth muscle*” [25]. These interpretations, however, were just speculative, as in those years it was generally thought that biological membranes were made of lipid bilayers coated with proteins at their cytoplasmic surface and associated with proteins or carbohydrates at their extracellular surface, but did not consider the existence of membrane channels [15,16].

Pentalaminar junctions were also reported in 1962 by Alan R. Muir and Alan Peters in capillary’s endothelial cells, astrocytes, and oligodendrocytes [26], but this study did not suggest that the junctions mediate direct cell–cell communication. Nonetheless, this is understandable because these cells are not electrically excitable, and no one in those years thought that non-excitable cells directly communicate with each other. A year later, Marilyn Farquhar and George Palade published superb images of cell junctions in several rat and guinea pig epithelia [27], and named all of them “*zonulae occludentes*” or “*tight junctions*”. They interpreted all of them as extracellular barriers to the para-cellular diffusion of molecules from lumenal to baso-lateral compartments of gland cells. However, this interpretation is surprising because these junctions were found in different areas of the cell surface, some far from the lumen. In addition, in spite of citing Karrer’s study, which showed similar junctional images in smooth muscle cells [24], they ignored Karrer’s view that these junctions might be involved in direct cell–cell communication. In retrospect, soon after it became obvious that while some of the junctions labeled “*zonulae occludentes*” [27] were indeed *zonulae occludentes* (tight junctions) functioning as extracellular barriers, other junctions, like those far from the lumen, were actually gap junctions.

### 4.2. Septilaminar Junctional Profiles

In the early 1960s, gap junctions and tight junctions (*zonulae occludentes*) were frequently confused because the techniques available in those years did not enable one to distinguish them, because all the electron micrographs displayed pentalaminar profiles. In addition, nobody was aware then that most neighboring cells directly communicated with each other.

**Figure 1:**
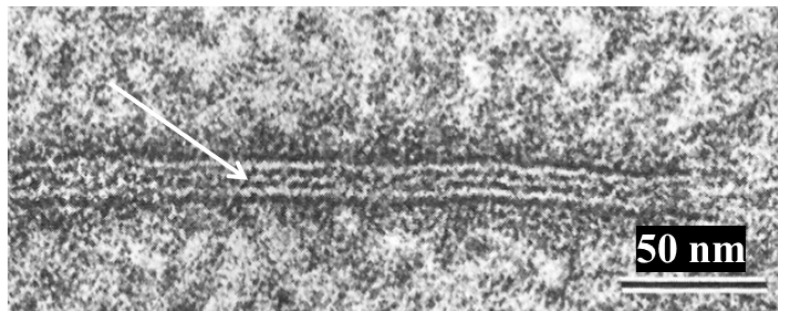
Cross-section profile of rat liver gap junction. The two membranes of the junction are separated by a 20–30 Å gap (arrow), creating a septilaminar profile (four electron-opaque and three electron-transparent lines). Adapted from ref. [32].

An important step forward took place in the mid-1960s when in-block staining with uranyl-acetate was first applied [28]; this technique was invented 8 years earlier for studying DNA-containing plasms [29]. By means of this technique, Jean Paul Revel and Morris J. Karnowsky reported that in some junctions, previously called tight junctions or *zonulae occludentes*, the membranes were in fact separated by a narrow extracellular space [30]. This space (called “gap”) generated septilaminar rather than pentalaminar profiles (Figure 1). These images prompted Revel to call these junctions “gap junctions” [31], while the term “tight junctions” or *zonulae occludentes* was used for pentalaminar junctions.

Revel and Karnowski proved the existence of an extracellular space at gap junctions by demonstrating that it was accessible to lanthanum hydroxide, an electron-dense extracellular tracer [30], used two years earlier by Carlos F. Doggenweiler and Samy Frenk for a different purpose [33]. The gap was, in fact, always described in invertebrate gap junctions because it is wider in these junctions: 50–75 Å [17], 40–85 Å [19], or 30–40 Å [21] in invertebrates and 20–30 Å [30] in vertebrates. Soon after, the gap in vertebrates was also shown to be permeable to potassium pyroantimonate in thin sections [34] and to horseradish peroxidase (MW 40,000) in isolated junctions [35,36].

### 4.3. The Puzzle of the 1960s

In the mid-1960s, scientists were puzzled because, while it was clear that cells directly communicated with their neighboring cells in both excitable and non-excitable cells, the structure thought to enable the exchange of ions and neutral molecules seemed more like a barrier than a permeable passageway. In fact, the pentalaminar membrane profile of mammalian junctions [22,23,24,25,26,27,37] resembled the profile of myelin, a structure functioning as an electrical insulator rather than a permeable cell–cell conduit. Consequently, Fritiof S. Sjöstrand and coworkers stated the following: “*… This would mean that the cell junction would represent a high ohmic resistance in the local current circuit*” [22]. This puzzle became even more difficult to solve when Revel and Karnowsky reported that the membranes of the junction were in fact separated by a 20–30 Å gap accessible to colloidal lanthanum and that the gap was not crossed by obvious bridges [30].

### 4.4. Hexagonal Arrays in Gap Junctions

The puzzle of early 1960s started being solved with Robertson’s report of polygonal arrays in junctions of goldfish’s Mauthner cells [38]. In samples fixed with potassium permanganate (KMnO_4_) dissolved in acetone, Robertson described hexagonal arrays in which the adjoined membranes, that he named “*synaptic discs*”, were seen in face-view sections. In Robertson’s words, “… *In frontal views a hexagonal array of close-packed polygonal facets is seen. These repeat at a period of ~95 Å. Each has a central dense spot <25 Å in diameter... The findings reported here are tentatively interpreted to mean that the honey-comb appearance represents a direct derivative of a macro-molecular pattern mainly localized in the outside lipoprotein or lipopolysaccharide-protein layer making the outer surfaces of unit membranes. The possibility must be entertained that the pattern extends all the way through the unit membranes as some micrographs might be taken to suggest. However, the slightest degree of tilt or wrinkling in the transversely sectioned synaptic discs could give such an effect even if the pattern is entirely confined to the central ~30 to 40 Å dense stratum.*” [38]. Despite publishing images showing that “... *the pattern extends all the way through the unit membranes…*”, Robertson still believed that the hexagonal arrays were just extracellular decorations. Indeed, his “*unit membrane*” [16] did not accept that any structure might cross the lipid bilayer, as he did not believe that membrane channels existed [39,40].

**Figure 2:**
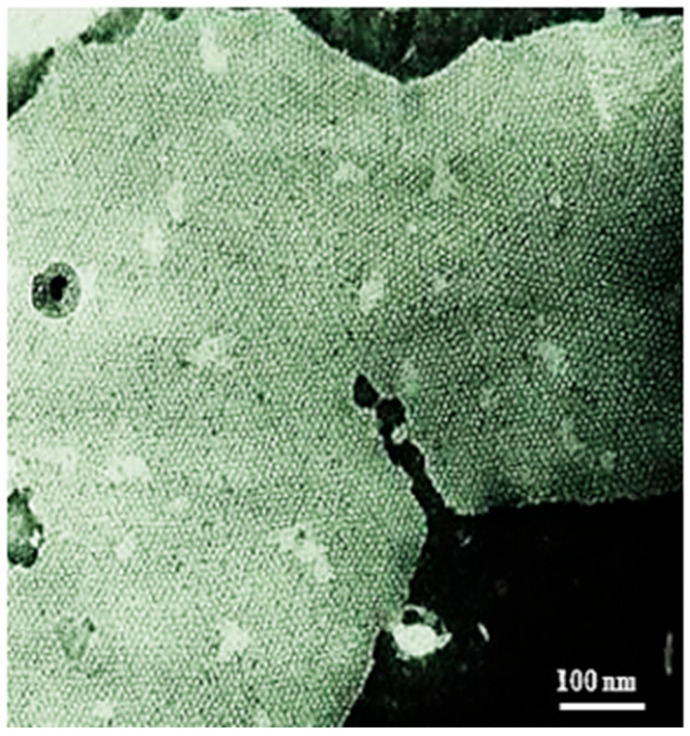
Isolated rat liver gap junction negatively stained with sodium phosphotungstate. The gap junctional particles (connexon channels) are aggregated in a hexagonal array at a center-to-center spacing of ~85 Å. Adapted from ref. [32].

Two years after Robertson’s publication [38], similar hexagonal arrays were reported by E. L. Benedetti and P. Emmelot in membranes isolated from rat liver and negatively stained with sodium phosphotungstate [41] (Figure 2). At first these authors believed that these arrays resulted from changes in the membrane’s lipid-phase caused by exposure to 37 °C [41]. Later, however, they realized that the arrays belong to gap junctions [41], as they learned that similar arrays were seen in the gap junctions of mouse liver and heart negatively stained with the extracellular tracer colloidal lanthanum [30]. Soon after, similar polygonal arrays were observed in various vertebrate and invertebrate gap junctions [21,34,35,42,43,44,45,46,47]. All of these studies reported images of hexagonal arrays, but there seemed to be a contradiction between Robertson’s data [38,48] and those of both Revel and Karnowsky [30] and Benedetti and Emmelot [41] because, while Robertson’s specimens were positively stained by the fixative potassium permanganate [38,48], the others were negatively stained by colloidal lanthanum [30] and sodium phosphotungstate [49], respectively. In the early 1970s we solved this puzzle because, by preparing acetone solutions of potassium permanganate, as Robertson did, we found that they in fact generate a precipitate of colloidal manganese dioxide (Peracchia, unpublished). Therefore, Robertson’s images were in fact those of negatively stained gap junctions, as the colloidal manganese dioxide must have diffused into the junctional gap and, being trapped there, it must have negatively stained the extracellular surfaces of the junctional membranes.

The structure first reported by Roberson was described as an array of electron-transparent “*facets*”, each displaying a central electron-opaque dot ~25 Å in diameter [38]; similar central dots were also reported by Revel and Karnowsky and Benedetti and Emmelot [30,41,49], but Brian W. Payton and coworkers were the first to suggest that the dots represent the openings of cell–cell channels filled with negative stain [42]. In their study, which also proved that a dye (Procion Yellow) diffuses across the septa of crayfish giant axons, they wrote that “… *The most likely site of passage of Procion Yellow between cell cytoplasms would then be in the center of the hexagonal regions outlined by the channels continuous with extracellular space*” [42]. However, this interpretation of the cell–cell channel location was just speculative because the images seen in negatively stained sections [30,41,42,49] only revealed the surface structure of the junctional membranes. Indeed, no one knew then whether the hexagonal arrays were superficial structures that decorated the membrane surface, projections of structures embedded in the membrane thickness, or structures that spanned the membrane thickness. Many of these puzzles began to be solved by the late 1960s and early 1970s when the powerful technique of freeze-fracture (also known as freeze-etching), invented in the late 1950s [50], started being applied to biological specimens [50,51,52,53].

### 4.5. Freeze-Fracture of Gap Junctions

The advantage provided by the freeze-fracture technique is that it enables one to view the internal structure of the membrane, as membranes oriented parallel or slightly oblique to the fracture plane split down the middle of the lipid bilayer, such that the intra-membrane structures associated with protoplasmic or exoplasmic membrane leaflets (P- or E-faces, respectively) become exposed. At first, the membrane-splitting interpretation [54,55] was met with skepticism, but it was soon proven by evidence that in red blood cells decorated either with ferritin [56] or with f-actin [57], these molecules were not seen on fractured surfaces but, instead, only appeared on un-fractured (etched) membrane surfaces.

**Figure 3:**
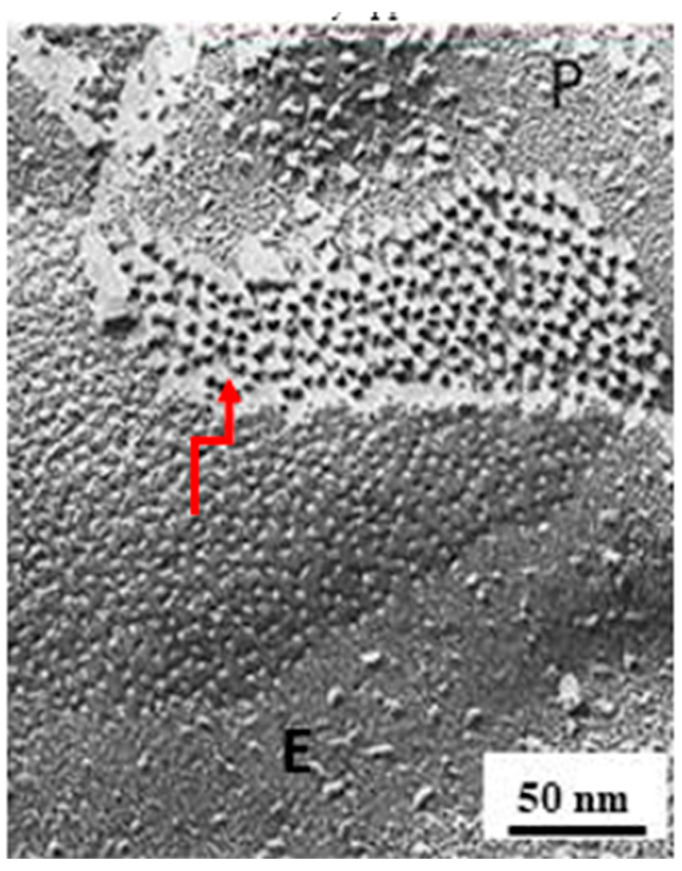
Freeze-fracture replica of a gap junction from an epithelial cell of rat stomach. The fracture plane steps down (red arrow) from the Exoplasmic face (E) to the Protoplasmic face (P). Particles (channels) and pits are randomly packed at a center-to-center spacing of ~10.4 nm, typical of coupled junctions. Adapted from ref. [61].

Freeze-fracture images of vertebrate gap junctions were first published in the late 1960s [43,54,55,56,58,59,60]. Two images were observed (Figure 3): one showed ~8 nm particles aggregated in polygonal arrays at ~10 nm center-to-center spacing on protoplasmic fractured faces (P-faces); the other showed somewhat similar aggregates of dimples on exoplasmic fractured faces (E-faces). Various interpretations of these images were proposed, but eventually all of them proved to be incorrect [21,47].

## 5. Early Models of Gap Junction Architecture

In their early independent studies, N. S. McNutt and R. S. Weinstein and J. P. Chalcroft and S. Bullivant did not believe that freeze-fractured membranes split down the middle [43,57], in spite of the fact that, three years earlier, Daniel Branton had produced compelling evidence for it [52]. In contrast, they believed that the images represented the structure of true external and internal membrane surfaces; in Bullivant’s words, “*The inner aspect of the plasma membrane is covered with a 90 Å centre-to-centre array of small depressions. Where this membrane is torn away, a less well ordered array of particles is seen in the extracellular space*” [59].

### 5.1. Model of Chalcroft and Bullivant

**Figure 4:**
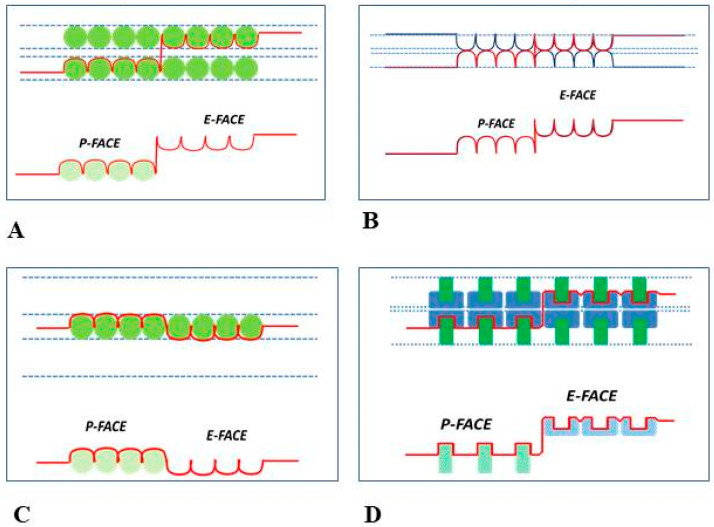
Early models of the architecture of gap junctions. Below the models are their freeze-fracture profiles (red line). (**A**). Chalcroft and Bullivant (1970) [57]. (**B**). Sommer & Steer (1969, 1972) [56,62]. (**C**). Spycher (1970) [54]. (**D**). McNutt and Weinstein’s (1969, 1970) [43,55]. Adapted from ref. [32].

Chalcroft and Bullivant, who produced the first double (complementary) replicas of gap junctions, believed that the fracture plane runs along one of its surfaces [57]; in their words, *“… in the gap junction, the fracture goes either near to or at the interface between membrane and cytoplasm, revealing particles within the membrane. …. The fracture goes around particles within the membrane, but we do not know whether it goes within the membrane or at the interface between membrane and cytoplasm when it is not in the region of particles”* [57]. Consequently, in their model (Figure 4A), the intra-membrane particles are shown to be totally embedded within the membrane, such that they would be unable to contact each other across the extracellular gap feature in obvious contradiction with evidence for direct cell-to-cell communication.

### 5.2. Model of Sommer and Steer

The model published by J. R. Sommer and R. J. Steer proposed that the junctional membranes are arranged in a honeycomb-like manner [56,62] such that the membranes would not contain intra-membrane particles but rather would display arrays of dimples, appearing as bumps on the P-face and dimples on the E-face (Figure 4B).

### 5.3. Model of Spycher

M. A. Spycher’s model proposed that the junctional membranes contain just one layer of particles located between the two membranes [54] (Figure 4C). However, this model would be inconsistent with data proving direct cell-to-cell communication.

### 5.4. Model of McNutt–Weinstein

A very complex model was published by N. S. McNutt and R. S Weinstein [43] (Figure 4D). In this model, while the fractured P-face was properly suggested to contain an aggregate of particles, the pits seen in the fractured E-face were thought to be images visible only where carbon-platinum is deposited on the fractured membranes at high angles. In contrast, the true E-face images were thought to be seen only where the carbon-platinum coated the membranes at low angles; these structures were named “*low-relief cobblestones*”, with very small central depressions [43]. The small (10–15 Å) depressions seen in the cobblestones were thought to be complementary images of the 70–100 Å P-face particles, which is impossible to accept due to their huge difference in size. A few years later, we correctly interpreted the “cobblestone” images in the opposite way, as we proved that the cobblestones with a very small central depressions were in fact dimples containing a small central bump [21,47] (seen in the following).

### 5.5. The Correct Model of Gap Junction Architecture

**Figure 5:**
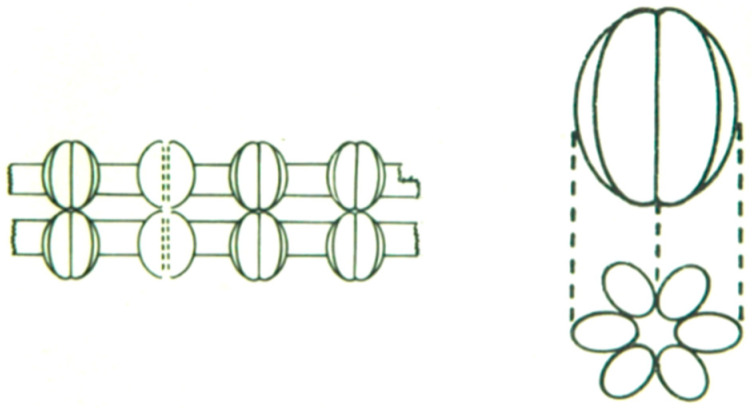
Correct model of gap junction architecture, based on ultrastructural images of gap junctions between septate axons of crayfish. The gap junction particles (channels) cross the thickness of the membrane, project from both surfaces of the membrane, are composed of six subunits, show small pits on both the cytoplasmic and the extracellular ends (channels’ openings), and bind to each other across the gap. Adapted from ref. [32].

In 1973, we published the first correct model of gap junction architecture (Figure 5) based on ultrastructural images of crayfish gap junctions studied by various techniques [21,47] (seen in the following).

## 6. The Gap Junction Channels Span the Thickness of the Membrane

**Figure 6:**
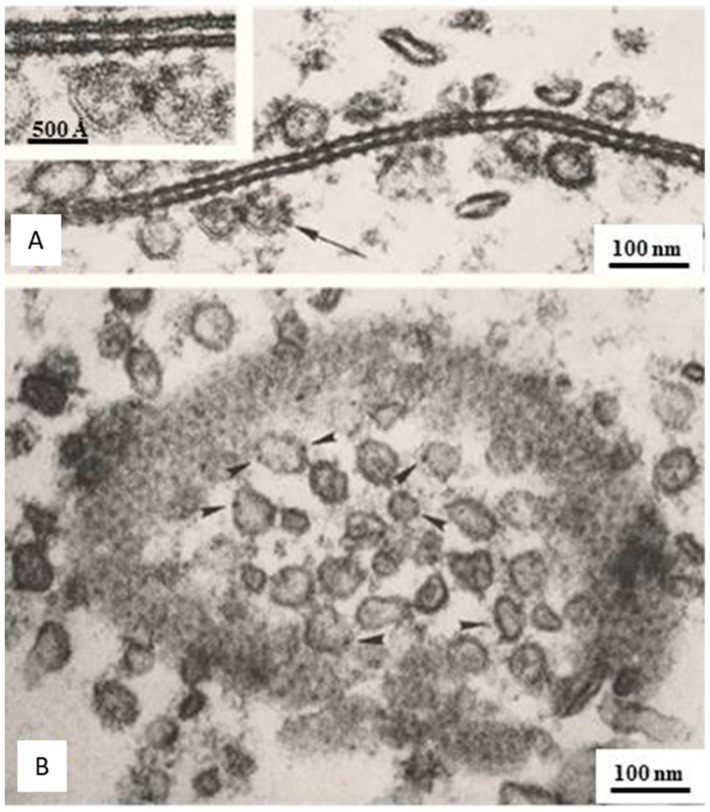
Gap junctions of crayfish lateral giant axons. In cross-sections (**A**), the junctions show a beaded profile representing electron-opaque particles (cell–cell channels) that protrude from both membrane surfaces and bind to each other across the gap. In face-view images (**B**), the channels are seen as electron-dense spots aggregated in a polygonal array spaced at ~200 Å center-to-center distance. Similar particles (hemichannels?) are also seen in adjacent vesicles (**A**,**B**), arrows and occasionally appear to interact with the junctional channels (**A**), inset. Adapted from ref. [21].

**Figure 7:**
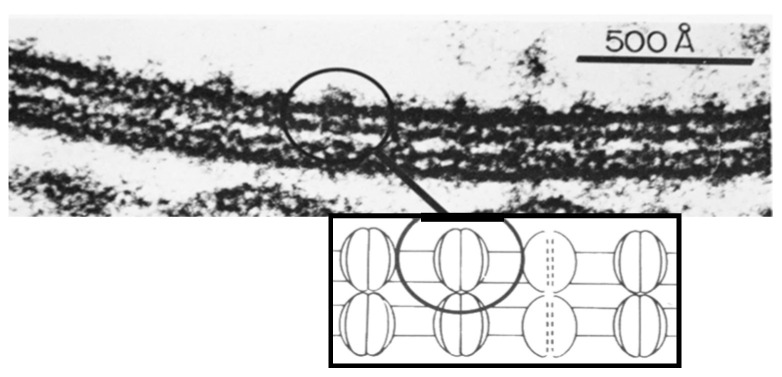
Cross-section profile of a crayfish gap junction showing a channel (circled) crossing the electron-transparent membrane layer. The channel’s center-to-center distance is ~200 Å. This confirms that the channels cross the thickness of the membrane. Adapted from ref. [21].

**Figure 8:**
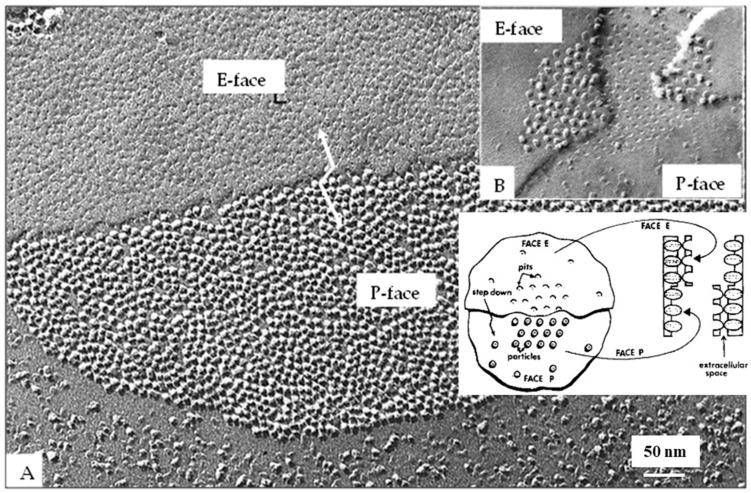
A vertebrate (**A**) and a crayfish (**B**) gap junction fixed in coupled cells and viewed by freeze-fracture electron microscopy. In both junctions the channels are aggregated in a disordered array. While in vertebrate junctions (**A**), rat liver, all connexons remain with the protoplasmic membrane leaflet (**A**), inset, appearing as particles and dimples on P- and E-faces, respectively (**A**), in crayfish gap junctions (**B**), most channels remain with the exoplasmic leaflet (**B**), inset, appearing as particles and dimples on E- and P-faces, respectively (**B**). Adapted from ref. [61].

**Figure 9:**
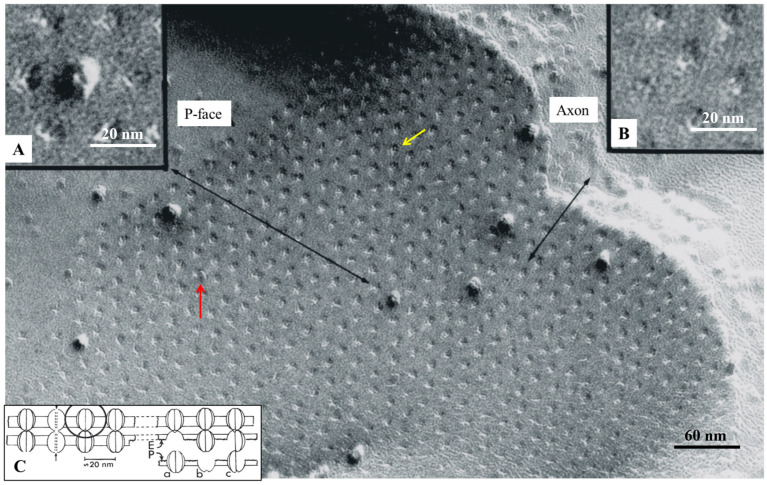
Freeze-fracture image of a crayfish gap junction coupling lateral giant axons. While most of the channels remain with the exoplasmic leaflet (**C**,b), a few remain with the protoplasmic leaflet (**C**,a), such that on the same fractured face one sees both the channels (**A**), center and the dimples (**B**) left by channels that had been fractured away. the P-face shows mostly dimples and a few particles. The particles are the extracellular half of the channels, and the dimples are the impression left by channels that have remained in the exoplasmic leaflet; they are the negative images of the cytoplasmic half of the channels. Since the membranes are fractured in the middle of the bilayer, these images prove that the channels cross the thickness of the membrane. Adapted from ref. [47].

In cross-sectioned junctions, images of channels spanning the membrane bilayer are often seen (Figure 6A and Figure 7) [21], but more convincing evidence was provided by freeze-fracture replicas [47]. Unlike vertebrate junctions in which all channels are fractured away with the protoplasmic leaflet, displaying particles on P-faces and pits on E-faces (Figure 3 and Figure 8A), in crayfish junctions, most channels remain associated with the exoplasmic leaflet (Figure 8B and Figure 9), while a few channels remain with the protoplasmic leaflet (Figure 9). Therefore, on the same fractured replica, one sees both channels and dimples left by channels that have been fractured away. Figure 9 shows an example of a P-face containing mostly dimples and a few channels (see model in Figure 9C). The particles are images of the extracellular surface of the channels, and the dimples are the impressions left by channels that have been fractured away, as they are the negative images of the cytoplasmic surfaces of the channels. Since the membranes split in the middle of the bilayer [52,53,63,64], these images (Figure 9) provided the first unequivocal evidence that the channels cross the membrane [21,47].

## 7. The Channels Project from Both Surfaces of the Membrane

Evidence for this is provided by cross-section images of junctional membranes, which display rows of electron-dense particles (innexon channels) emerging from both surfaces of the membrane (Figure 6A and Figure 7) [21].

## 8. The Channels Are Made of Six Radially Arranged Subunits (Hexamers)

**Figure 10:**
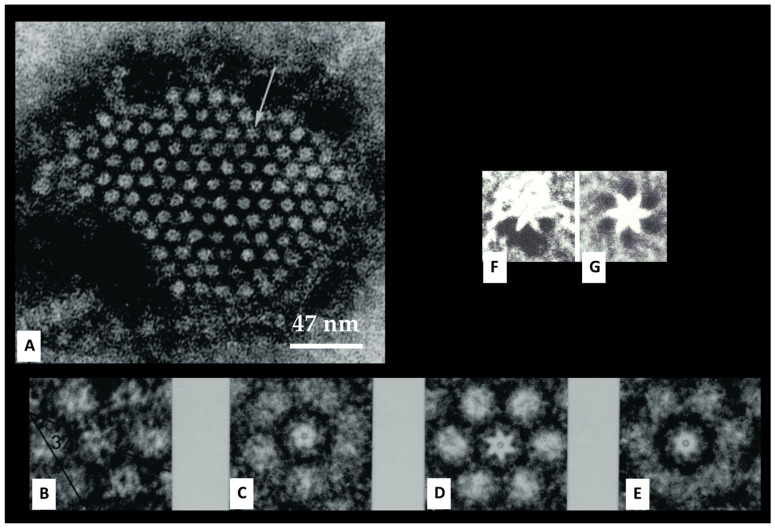
Crayfish gap junction isolated and negatively stained with phosphotungstic acid (**A**). The arrow (**A**) points to a channel that displays its hexameric structure. The six radially arranged subunits are also seen in freeze-fracture replicas (**F**) and in thin-sectioned junctions stained with colloidal lanthanum (Figure 11A(a)). Evidence of six subunits is confirmed by the method of Markham and coworkers, as just a 6-step rotation creates a clear image of 6 subunits (**D**,**G**), and Figure 11A(b). In contrast, 5 (**C**) and 7 (**E**) step rotations do not display images of subunits. In (**A**), the channels are aggregated in a hexagonal array at a center-to-center distance of ~150 Å. Adapted from ref. [47].

**Figure 11:**
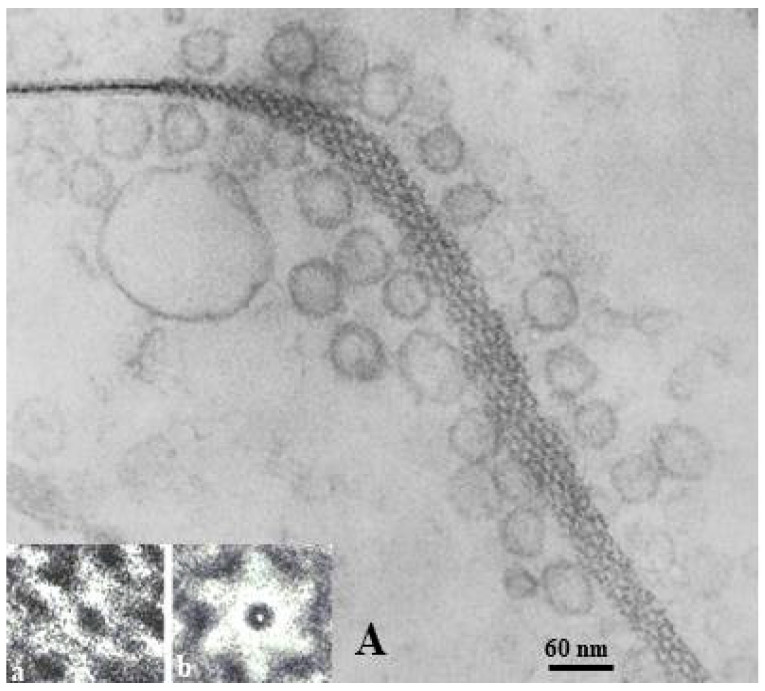
Crayfish gap junction viewed in thin sections negatively stained with colloidal lanthanum (**A**). The channels are seen as electron-transparent spots encircled by the electron-dense stain that fills the gap. Some of the channels show a central electron-dense spot (**A**,**a**) that represents the location of the channel’s pore. Faint images of six pointed stars (**A**,**a**) and the channel’s subunits (innexons) are confirmed by the six-step rotation of the method of Markham and coworkers (**A**,**b**). Adapted from ref. [21].

Gap junctions of crayfish axons provided the first evidence of the hexameric architecture of innexons/connexons, which was based on images of six pointed stars [2,6] in channels seen in fragments of isolated gap junctions stained negatively with phosphotungstic acid (Figure 10A,B) [6], in channels viewed in thin sections of axons treated with the extracellular tracer colloidal lanthanum (Figure 11A(a)) [2], and in channels seen in freeze-fractured specimens (Figure 10F). The number of subunits was confirmed by the method of Markham and coworkers [65], a multiple-rotation photographic-exposure, which proved that only six-step rotations generated clear images of six subunits (Figure 10D,G and Figure 11A(b)). The existence of subunits was also indirectly confirmed by evidence of small particles located at one side of P-face dimples (Figure 9, red arrow, and C,c) [6]. This indicates that the fracture occasionally splits some channels along the axis perpendicular to the membrane plane, leaving some subunits (innexins) in place and fracturing away the other subunits (Figure 9Cc). Several years later, the hexameric composition of innexon/connexon channels was confirmed in vertebrate gap junctions as well [61,66,67].

## 9. The Channels Display Dimples on the Cytoplasmic and Extracellular Ends, Representing the Cytoplasmic and Extracellular Channel Opening, Respectively

The electron-opaque ~20 Å spots seen at the center of negatively stained gap junction particles (Figure 9A, Figure 10 and Figure 11A(a)) [47] were previously suggested to represent the location of the channel pore [42,43]. However, it was unclear then whether they represented the location of cell–cell channels. Even freeze-fracture images of central dimples seen in particles of vertebrate gap junctions could not prove that they were indeed the openings of cell-to-cell channels as it was not known whether similar dimples existed at the cytoplasmic end of the particles. In fact, only the extracellular connexon’s end is exposed in freeze-fracture replicas of vertebrate gap junctions (Figure 3 and Figure 8A).

More convincing evidence of the gap junction channel’s location was provided by freeze-fracture images of crayfish junctions, because here some of the fractured particles remain attached to the protoplasmic leaflet (P-face; Figure 9), while others remain with the exoplasmic one (E-Face; Figure 8B) [47]. Indeed, images of central dimples are seen in particles exposed on both P-face (Figure 9A) and E-face replicas [47]. Moreover, dimples visible on P-face replicas after particles had been fractured away showed obvious images of central bumps (Figure 9, yellow arrow, and B), which confirmed that central dimples were also present at the cytoplasmic end of the particles. The small central bumps (Figure 9B) represent cytosolic medium filling the cytoplasmic vestibule (mouth) of the channels [47].

### Freeze-Fracture Images of Central Pits on the Cytoplasmic and Extracellular Ends Match the Electron-Dense Spots Seen in Sections Negatively Stained as Well as in Junctions Isolated, Demonstrating the Location of the Channel

**Figure 12:**
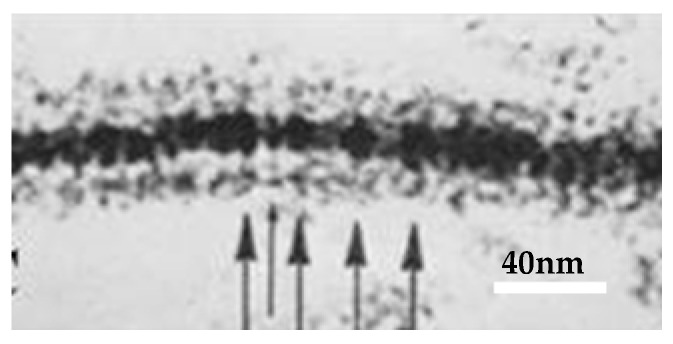
Cross-sectioned junction of a crayfish axon negatively stained with colloidal lanthanum. The electron-dense line that crosses a portion of the junctional profile at the center of adjoined channels (small arrow) represents the longitudinal view of the cell–cell channel which is partially occupied by lanthanum; note that lanthanum does not reach the cytoplasmic surface of the channel, suggesting that the channel is blocked at its cytoplasmic end (uncoupled junction). The spaces between neighboring channels filled with lanthanum are indicated by the larger arrows. The channels repeat at a center-to-center distance of ~150 Å. Adapted from ref. [21].

In isolated junctions negatively stained (Figure 10A,B) [47] as well as in junctions thin-sectioned and lanthanum-stained (Figure 11A(a)) [21], many channels display central electron-opaque spots, indicating the location of the channel’s pore. In cross-sectioned junctions (Figure 12), an electron-opaque line is seen spanning a portion of the junction profile at the center of adjoined innexons. This line represents the longitudinal image of the cell-to-cell channel partially occupied by lanthanum (Figure 12 small arrow) [21], while the larger arrows indicate the extracellular spaces filled with lanthanum that separate neighboring channels. Note, however, that the lanthanum that occupies the channel’s pore does not reach the cytoplasmic end of the channel (Figure 12 small arrow), suggesting that the pore is blocked at the cytoplasmic end (uncoupled junction).

## 10. The Channels Are in Register and Are Linked to Each Other Across the Gap

For channels to function as cell–cell pathways insulated from the extracellular solution, it is essential that their structures (innexons/connexons) are in register and are linked to each other for bridging the extracellular gap. Unequivocal evidence for this was provided by cross-sectioned images of crayfish junctions (Figure 6A and Figure 7) [21].

## 11. Calmodulin (CaM) Is an Accessory Protein of Connexin/Innexin Channels

CaM is linked to gap junction channels and plays a major role in channel gating, channel aggregation and channel formation; rev. in [68].

## 12. Channel Arrays in Coupled and Uncoupled Gap Junctions

**Figure 13:**
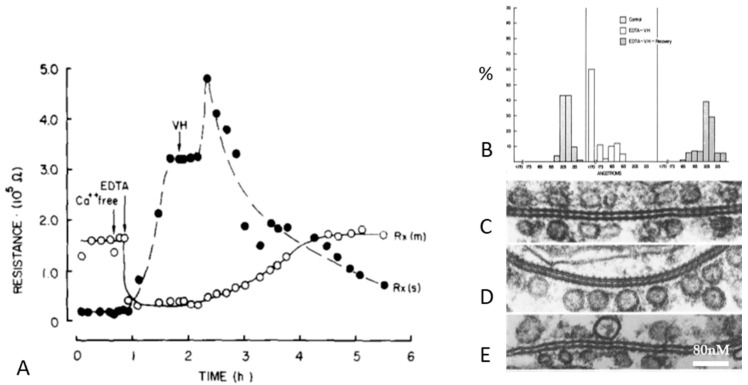
Crayfish gap junctions. (**A**), Junctional (septal, Rxs) and membrane (Rxm) electrical resistances monitored during uncoupling by ethylenediaminetetraacetic acid (EDTA) followed by Van Harreveld’s (VH) saline. (**B**), Histogram of channels’ center-to-center distances in thin-sectioned electron micrographs; the channels’ center-to-center distance is ~205 Å in coupled and recoupled junctions and ~170 Å in uncoupled junctions. (**C**), control junction; (**D**), uncoupled junction; (**E**). recoupled junction. (**C**–**E**), Electron micrographs of cross-sectioned gap junctions. Adapted from ref. [66].

**Figure 14:**
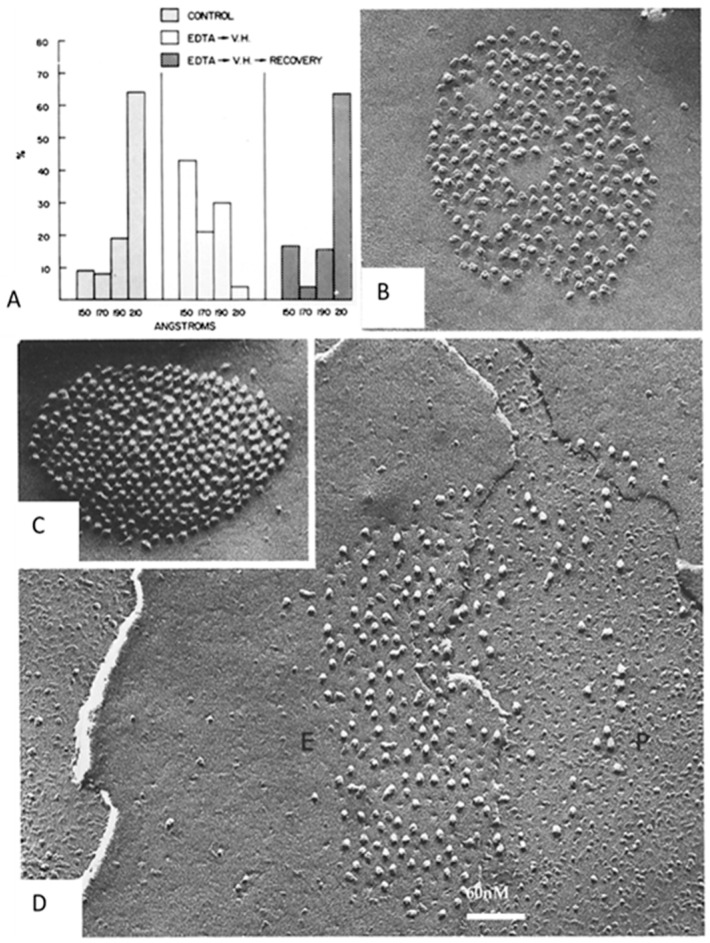
Crayfish gap junctions. (**A**), frequency histogram of channels’ center-to-center distances in junctions fixed in coupled, uncoupled and recoupled conditions. Most of the channels’ center-to-center spaces, which is 210 Å in coupled and recoupled junctions and drops to 150–170 Å in uncoupled junctions. (**B**), freeze-fracture replica (FF) of control junction. (**C**), FF of junction uncoupled by ethylenediaminetetraacetic acid (EDTA) followed by Van Harreveld’s saline (VH). (**D**), FF of recoupled junction. P, protoplasmic fracture face. E, exoplasmic fracture face. Adapted from ref. [66].

In the early 1970s, we noticed that the way gap junction channels are aggregated varies significantly, as the channels appeared aggregated either in fairly disordered/loose arrays or in tight crystalline hexagonal arrays [2,6]. In well-coupled crayfish axons, the channels were loosely packed and spaced at ~200 Å center-to-center distance in both thin sections (Figure 13C) [2] and freeze-fracture replicas (Figure 14B) [6]. In contrast, in isolated negatively stained gap junction fragments, the channels were tightly packed in crystalline arrays and were spaced at 150–155 Å center-to-center distance (Figure 10A) [6].

**Figure 15:**
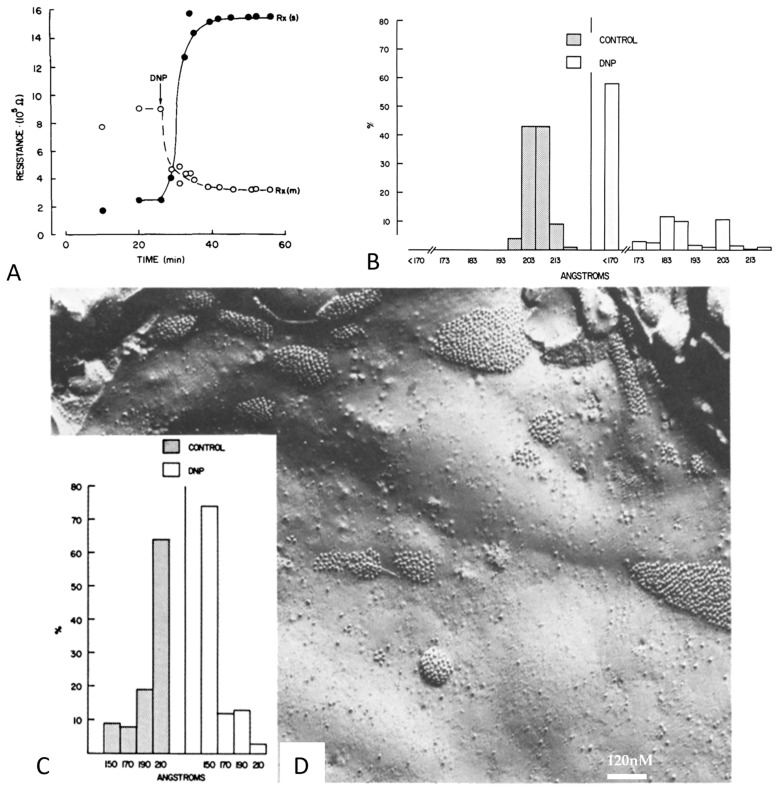
(**A**), time course of crayfish junctional electrical resistance Rx(s) (filled circles), and membrane resistance Rx(m) (open circles) during exposure to 2,4 dinitrophenol (DNP). With DNP, Rx(s) increases from ~3 × 105 Ω to ~15.5 × 105 Ω. (**B**), histogram of center-to-center channel spacings measured in cross-sectioned junctions of control and DNP-treated axons; with DNP, the channels’ center-to-center distance drops from ~210 Å to ~170 Å. D. (**C**), histogram of channel spacings in freeze-fractured specimens of control and DNP-treated junctions; with DNP, the channels’ center-to-center distance drops from ~210 Å to ~170 Å. (**D**), freeze-fracture of gap junctions (E face) in a ganglion treated with DNP and fixed in uncoupled state. Most junctions are dome-shaped (see in the following) and display tightly packed channels at an average center-to-center distance of 150–155 Å. Adapted from ref. [66].

Based on these differences, we suggested that the different types of channel aggregation might reflect different states of channel permeability [21,47]. In the mid-1970s, we tested this hypothesis by studying, in crayfish axons, the effects of uncoupling treatments on junctional electrical resistance (Rj) and gap junction architecture (Figure 13, Figure 14 and Figure 15) [69,70,71].

Rj and gap junction architecture were first studied in crayfish lateral giant axons at rest or uncoupled either by treatment with ethylenediaminetetraacetic acid (EDTA) followed by Van Harreveld’s saline (crayfish saline; Figure 13 and Figure 14) or by exposure to 2,4 dinitrophenol (DNP, a metabolic inhibitor) (Figure 15) [69]. The junctions were viewed in both thin sections (Figure 13) and freeze-fracture replicas (Figure 14 and Figure 15). Most junctions fixed when Rj was high showed tightly packed channel arrays (Figure 13, Figure 14 and Figure 15), while in controls and in samples processed after full recovery of normal channel permeability, most junctions displayed loosely arranged channels (Figure 13, Figure 14 and Figure 15). The changes in channel array were thought to reflect conformational rearrangements of channel structure reflecting the closure of the channel’s pore [69].

**Figure 16:**
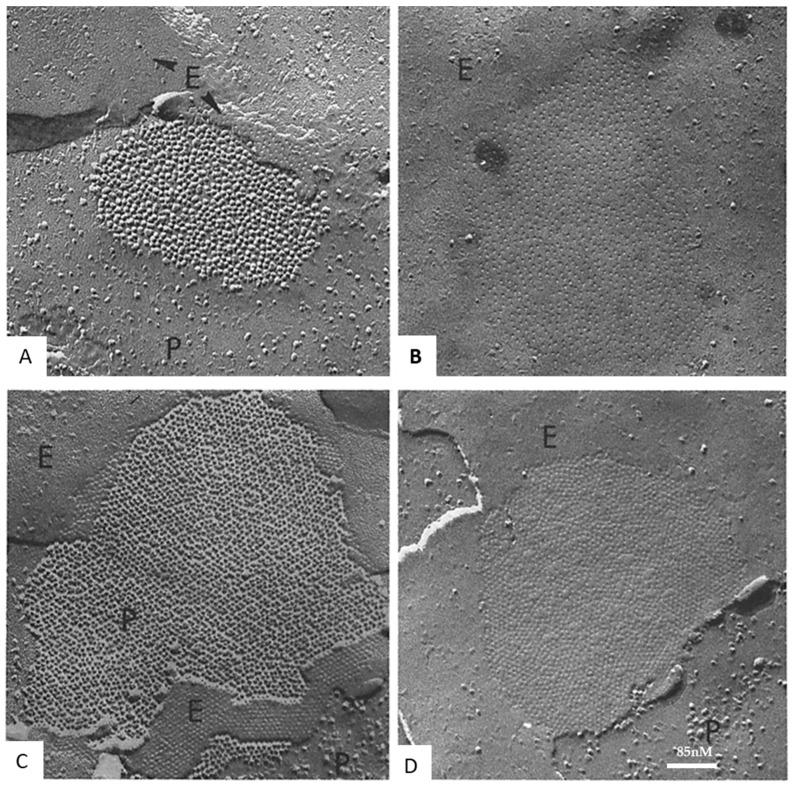
Freeze-fracture replicas of rat stomach gap junctions, P = P face; E = E face. (**A**,**B**), control junctions from samples kept for 1 h in a well-oxygenated Tyrode’s solution. Channels (**A**) and channel dimples (**B**) are irregularly arranged at an average center-to-center distance of ~10.2 and ~12.5 nm, respectively. (**C**,**D**), junctions from samples kept for 1 h in 2,4 dinitrophenol (DNP). Channels (**C**) and channel dimples (**D**) are hexagonally aggregated at center-to-center distances of ~8.58 nm (**C**), and ~8.6 nm (**D**). Adapted from ref. [75].

Soon after the crayfish study [69], similar changes in channel arrays were seen in gap junctions of rat stomach (Figure 16) and liver subjected to uncoupling treatments such as hypoxia or exposure to DNP [65,70,71,72]. In these cells, disordered arrays with channels spaced at a center-to-center distance of 10–11 nm (Figure 16A,B), typical of control junctions, changed with uncoupling treatments into hexagonal arrays with channels spaced at a center-to-center distance ~8.5 nm (Figure 16C,D).

Tight crystalline channel arrays were also reported in mechanically injured cardiac cells [61,66] as well as in junctional membranes split apart by perfusion with hypertonic sucrose [70]. This is reasonable because the membranes of junctions split apart have high electrical resistivity [67,73]. Channel crystallization was also reported by Goodenough and Gilula in gap junctions of cells exposed to hypertonic sucrose [74], but neither in this study nor in the cardiac studies [61,66] was channel crystallization thought to reflect changes in channel permeability.

## 13. Calmodulin Role in Gap Junction Channel Gating and Aggregation

Numerous studies have reported that high nanomolar to low micromolar cytosolic calcium concentrations [Ca^2+^]_i_ activate cell–cell channel gating in both vertebrate and invertebrate cells [76,77,78,79,80,81,82,83,84,85,86,87,88,89,90,91,92,93,94,95,96,97,98,99]. Based on this high Ca^2+^-gating sensitivity, it is not only clear that cytosolic calcium (Ca^2+^_i_) is a fine regulator of cell-to-cell channel permeability, but also that Ca^2+^-modulated proteins like CaM mediate the Ca^2+^_i_-dependent channel gating. Indeed, gap junction channel proteins (connexins and innexins) do not have highly sensitive Ca^2+^-binding sites like the EF-hand domains typical of CaM-like proteins [100,101,102].

The role of CaM in gap junction channel gating has been confirmed by multiple studies that have tested the effect of treatments such as exposure to CaM blockers, CaM expression-inhibition, overexpression of CaM mutants, CaM co-localization with gap junctions, and in vitro binding of CaM to connexins (Cx) or synthetic peptides mimicking CaM-binding sites of connexins, among others [32,103,104,105,106,107,108,109,110,111,112,113,114,115,116,117,118,119,120,121].

In 2000, we have proposed a CaM-mediated “cork-type” gating model [122]. The model, named “Ca-CaM-Cork” gating, envisions physical blockage of the channel’s mouth by a CaM lobe (N-lobe?), likely to be combined with conformational changes in connexins induced by Ca^2+^-CaM binding to Cx sites. Ca^2+^-CaM gating is only reversed by the return of cytosolic Ca^2+^ concentration ([Ca^2+^]_i_) to resting values.

A recent study on myocardial ischemia–reperfusion injury causing arrhythmia [123] has tested in isolated-perfused rat heart and in Cx43-expressing H9c2 cultured cardiac cells, the effect of a peptide called SP15, which mimics the CaM-binding site of a Cx43’s cytoplasmic loop sequence known to bind CaM, in an attempt to restore gap junction function for mitigating reperfusion arrhythmias. The peptide SP15 matches the CaM-binding sequence 144–158 (KVKMRGGLLRTYIIS), which is in the second half of Cx43’s cytoplasmic loop (CL2). The administration of SP15 encapsulated into liposomes reduced the interaction between Cx43 and CaM and inhibited arrhythmia. These data indicate that inhibiting CaM or interfering with the interaction between CaM and Cx43 can promote the opening of gap junctions and restore their function. To our knowledge, this is the first example which demonstrates that by manipulating the Ca-CaM-Cork gating mechanism, one may succeed in treating some pathological conditions in which cell–cell coupling needs to be restored. Furthermore, this study confirms once more the validity of the Ca-CaM gating mechanism.

There is plenty of evidence for the presence of a CaM-binding site at CL2 [90,115,116,119,124,125,126]. Particularly relevant to the study of Wang and coworkers [123] is the report by Wei and coworkers [126], which demonstrated that the CL2’s CaM-binding peptide of Cx50 prevents Ca^2+^/CaM-induced gating of gap junction channels in N2a cells expressing the mutant Cx43-M257. This indicates that peptides mimicking the CaM bind domain, regardless of which Cx they belong to, act non-specifically by binding activated CaM in gap junctions as well as in the cytoplasm. Therefore, CaM-binding peptides that mimic the CL2’s CaM-binding site do not selectively inhibit the Ca-CaM blocking action of the source Cx gap junctions, but act by inhibiting the CaM-sensitive gating of any connexin/innexin as well as the function of other CaM-activated proteins.

In addition to the CaM-mediated regulation of channel permeability, there is evidence for the roles of phosphorylation/dephosphorylation, nitric oxide, hydrogen sulfide or carbon monoxide, acetylation, methylation or ubiquitination, and transjunctional voltage gradients; for a review, see [127]. Gap junction channels are also gated in response to cytosolic acidification, but based on several studies, including our own, we believe that low pH_i_ act via an increase in [Ca^2+^]_i_; this is reviewed in [104,128].

## 14. What Causes Different Channel Aggregations in Gap Junctions?

**Figure 17:**
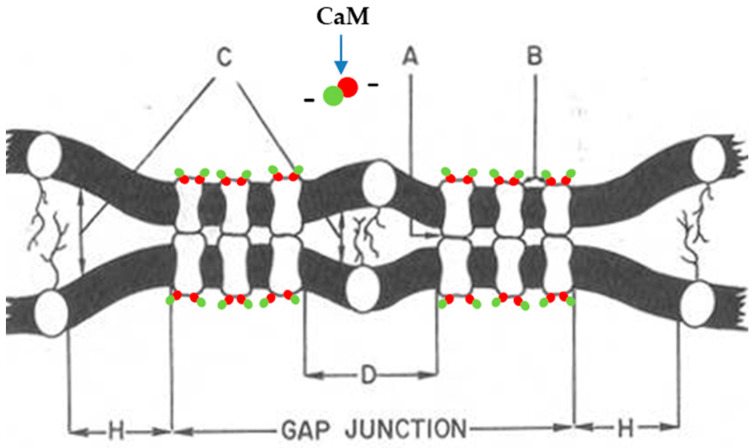
Model of forces involved in gap junction channel aggregation. Three forces are likely to be involved: a binding force A linking hemichannels across the gap; a repulsive force B likely to be caused by the repulsion of negatively charged CaM molecules; and a repulsive force C in peri-junctional mem-branes regions (particle-free halo, H) that forces channels to be aggregated. Area D corresponds to freeze-fracture images of smooth (~65 nm) disks seen in some junctions of sheep’s cardiac Purkinje fibers (see Figure 18). Adapted from ref. [75].

**Figure 18:**
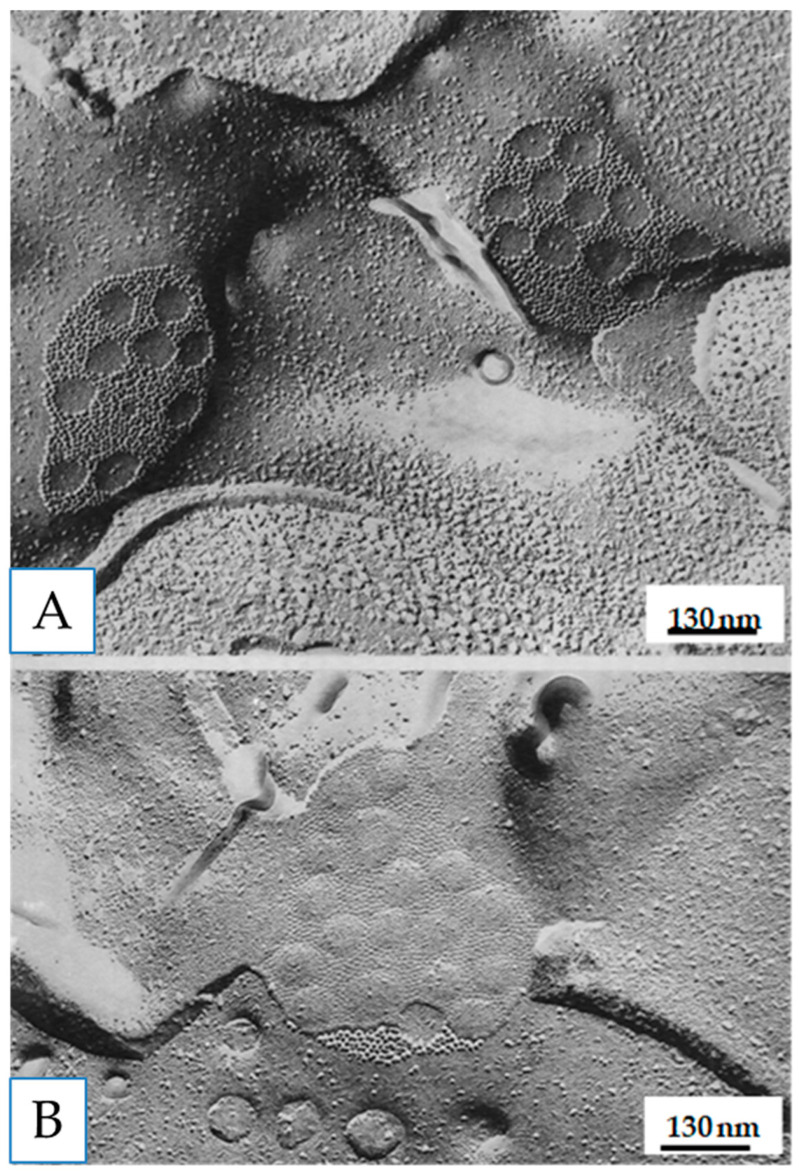
Images of smooth (~65 nm) disks in freeze-fractured gap junctions of sheep’s Purkinje fibers (**A**), P-face, and (**B**), E-face, containing one or two particles at their center (**B**). The central particles are believed to be glycoproteins trapped within the gap junction (see Figure 17, “C”). Adapted from ref. [75].

Still vague is our knowledge of what maintains gap junction channels aggregated into plaques and of the mechanism by which uncoupling treatments cause channels to aggregate more tightly into crystalline (hexagonal) arrays. We have proposed that the loose/disordered aggregation of gap junction channels, seen in well-coupled cells (Figure 3, Figure 8 and Figure 9), reflects the balance among three forces: (1) a linking force (Figure 17, “A”) that binds connexons/innexons across the gap, maintaining the junctional membranes linked to each other; (2) a repulsive force (Figure 17, “B”) between channels within the membrane; and (3) a repulsive force (Figure 17, “C”) between peri-junctional membranes that keeps channels aggregated [75]. Based on present knowledge of connexin/innexin sequences, it is clear that force “A” (Figure 17) results from the interconnection of the extracellular loops across the gap, while force “C” reflects the existence of glycoproteins which force neighboring membranes to be separated by 120–150 Å. Consistent with this is evidence that in some junctions, particle-free disks ~65 nm in diameter, displaying one or two particles at their center, can be seen in freeze-fracture replicas (Figure 18) [75]. Most likely, the central particles are glycoproteins trapped within the junction, whose oligosaccharide chains maintain the separation of junctional membranes and keep aggregated gap junction channels. Indeed, as predicted, the disks’ surface is concave on P-faces (Figure 18A) and convex on E-faces (Figure 18B). The particle-free halo surrounding the junctions (Figure 17, “H”) is also likely to result from the presence of glycoproteins that surround the junction [75].

The nature of forces “A” and “C” is obvious, while that of force “B” (Figure 17) and the mechanism by which loose channel arrays switch to tightly crystalline and the mechanism that causes hexagonal channel arrays with a rise in [Ca^2+^]_i_ is still unclear. Our hypothesis is that the loose/disordered channel aggregation results from the presence of CaM molecules bound to connexins in each hemichannel, which would be expected to repel each other due to their negative charges (Figure 17, “B”).

**Figure 19:**
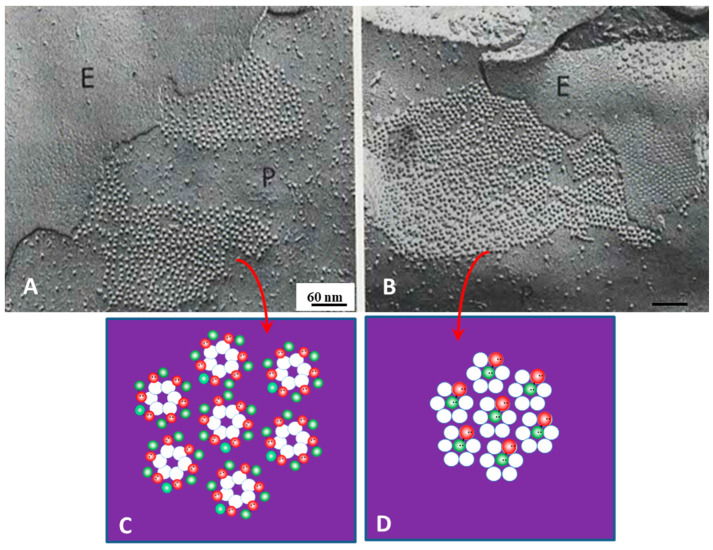
Images of freeze-fractured rat stomach’s gap junctions fixed either in a well-oxygenated Tyrode solution ((**A**), coupled junction) or in the same solution containing the calcium ionophore A23187 ((**B**), uncoupled junction). In coupled junctions (**A**), channels and channel dimples are loosely aggregated at an average center-to-center distance of 10.4 nm (**A**). In contrast, in uncoupled junctions (**B**), they are arranged in crystalline (hexagonal) arrays at an average center-to-center distance of ~8.6 nm. Our view of the mechanism causing the different particle arrays is shown in (**C**,**D**), respectively. While in coupled junctions (**A**), the particles (channels) are believed to be kept separated by the repulsive force of negatively charged CaM molecules (**C**), in uncoupled junctions (**B**) most of the CaM molecules, except the gating one, are believed to have become disconnected (**D**), allowing the channels to pack in tight hexagonal arrays. E = Exoplasmic face. P -= Protoplasmic face. (**A**,**B**), adapted from ref. [69].

**Figure 20:**
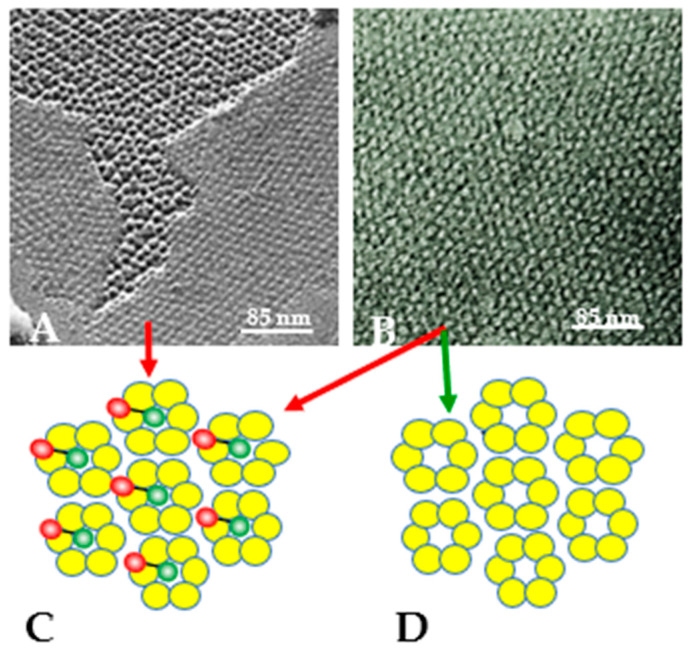
Gap junctions with channels in crystalline/hexagonal arrays. (**A**), freeze-fracture replica of a rat stomach gap junction fixed in uncoupled state. (**B**), gap junction isolated from rat liver and negatively stained with phosphotungstic acid. In both channels, center-to-center distance is ~ 8.5 nM. Our view is that in uncoupled junctions (**A**), only the gating CaM molecule is maintained (**C**), while in the isolated junction, the CaM molecules may or may not be washed away ((**C**,**D**), green and red arow, respectively) depending on the isolation procedure. Adapted from ref. [61].

We believe that in well-coupled junctions (Figure 19A), a CaM molecule is bound to each connexin/innexin of a connexon/innexon by the C-lobe, while the N-lobe is unbound. If so, there would be six free CaM lobes per connexon/innexon (Figure 19C)—twelve per cell–cell channel. Since CaM lobes are negatively charged, they would repel each other within and between channels, causing the channels to stay separated from each other (Figure 19A,C). In coupled junctions, the channels’ center-to-center distance is ~100 Å in vertebrates and ~200 Å in crayfish axons, and the channel arrays are loose and fairly disordered (Figure 19A). With a rise in [Ca^2+^]_i_ (Figure 19B), all CaM molecules but the gating one may become detached from connexins/innexons (Figure 19D), resulting in the elimination of the repulsive force “B” (Figure 17). If this were the case, the channels would become tightly packed into crystalline (hexagonal) arrays (Figure 19B,D), interacting with each other mostly hydrophobically. This might be the reason why in isolated junctions, the channels remain tightly bound to each other (Figure 10A and Figure 20B), even though the samples are subjected to harsh isolation procedures.

The obvious question is this: what could cause CaM molecules to be detached from connexins at high [Ca^2+^]_i_? Over two decades ago, Black and coworkers [129] made a genetically encoded fluorescent biosensor to determine the intracellular concentration of Ca^2+^-free (apo-CaM) and Ca^2+^-activated (holo-CaM) CaM. Their research demonstrated that in a human kidney cell line expressing the biosensor, [CaM]_i_ at rest is 8.8 ± 2.2 µM. A rise in [Ca^2+^]_i_ induced by either treatment with agonists, store-operated Ca^2+^ release, or application of Ca^2+^ ionophores, induced a drastic Ca^2+^-dependent drop in available free CaM molecules, causing in a drop in [CaM]_i_ to ≤200 nM. This is caused by drastic buffering of free CaM resulting from excess number of CaM-binding sites. The same data were reported in rabbit cardiac myocytes, with a rise in [Ca^2+^]_i_. CaM was reversibly transferred into the nucleus in both permeabilized and intact myocytes [130]. This study also proved that with a rise in [Ca^2+^]_i_, CaM moves from the Z-line to Ca^2+^-CaM binding sites of greater affinity. The same phenomenon might apply to gap junctions, a likely scenario being that with a great increase in [Ca^2+^]_i_ non-gating CaM, molecules likely to be bound to the connexins/innexins by one lobe are freed (Figure 20A,C), as other cytoplasmic CaM-binding sites with higher affinity to Ca^2+^-CaM successfully compete for CaM-binding.

In isolated junctions, the gating CaM may or may not remain associated with the junction depending on the isolation procedure (Figure 20B–D). Indeed, in the early 1980s, Makowski et al. reported that crystalline (hexagonal) gap junctions isolated from mouse liver were in high resistance configuration (closed channels) [131,132,133]. The channels’ gated condition was proven by the channels’ impermeability to sucrose, as the channels were blocked at both cytoplasmic ends by a small particle [131]. Significantly, the blocking particle had a spherical shape and was approximately 30–35 Ǻ in diameter [133], which is the actual size of a CaM lobe (Figure 20C). Similar blocking particles were seen in isolated Cx26 gap junctions [134]. On the other hand, a recent study [1,135] has reported that the channel’s pore is not obstructed (Figure 20D, green arrow) in gap junctions isolated by their procedure, suggesting that in this case, the gating CaM molecule might also have been washed away.

## 15. Dome-Shaped Gap Junctions

**Figure 21:**
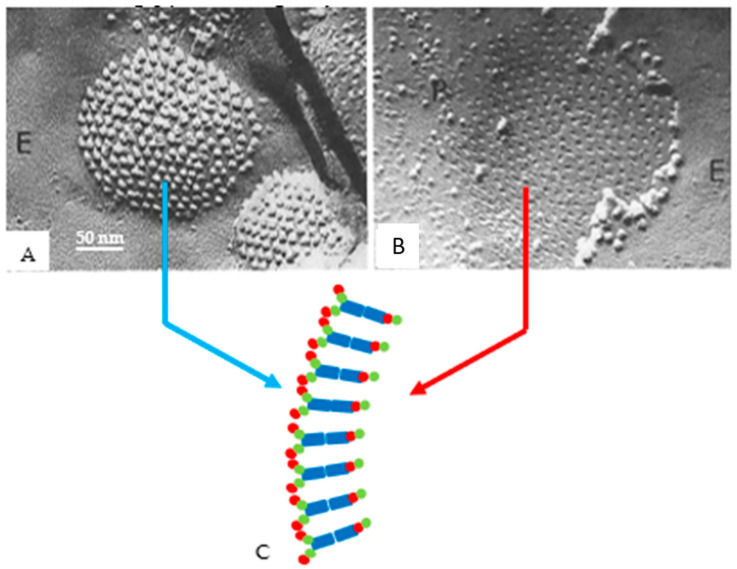
In uncoupled cells of crayfish axons, some junctions have a “dome-like” shape. Perhaps, in these junctions the channels are gated unilaterally, and five out of six calmodulin (CaM) molecules might have been detached just from the concave surface of the junction ((**B**), gated side), while at the convex surface, some or most of the CaM molecules are still attached to innexins ((**A**), ungated side); unilateral uncoupling? A very schematic hypothetical model of these junctions is shown in (**C**). E and P = Exoplasmic and Protoplasmic fracture faces, respectively. Adapted from ref. [66].

With a rise in [Ca^2+^]_i_, some crayfish gap junctions acquire a “dome-like” shape (Figure 15D and Figure 21A,B) [69]. Perhaps, in these cases, the junctional channels are gated unilaterally, so that five out of six CaM molecules might have been disconnected from connexons/innexons just from the concave surface (gated side), while most of the CaM molecules are still attached to connexins at the convex surface (ungated side) (Figure 21C). If this were the case, one would expect the junction to bend, assuming a “dome-like” appearance. This could also represent an early phase of gap junction retrieval, followed by gap junction internalization and the formation of intracellular annular gap junctions in the process of being degraded. Significantly, with prolonged exposure to uncouplers, the number of annular gap junctions is greatly increased [136].

## 16. Summary and Conclusions

In summary, this review has first addressed the complex history of gap junction architectural models and the major characteristics of channel ultrastructure. The following section has detailed our hypotheses of the relationship between different channel arrays and the cell–cell channel permeability status. Our view is that disordered (random) channel arrays correspond to gap junction channels of well-coupled cells, in which the channels are kept separated by the negatively charged CaM molecules. Tight, crystalline (hexagonal) channel arrays in intact cells are believed to reflect gated channels of uncoupled cells, in which only the gating CaM molecules remain associated with gap junction channels. In crystalline (hexagonal) channel arrays of isolated junctions, the gating CaM molecule may or may not be washed away, depending on the isolation procedure used. Dome-shaped gap junctions might reflect unilaterally gated gap junction channels. While we feel that CaM plays a role in determining different channel arrays, it should be understood that, at this stage, this is just a hypothesis and other mechanisms may be involved in the process.

## Data Availability

No new data were created or analyzed in this study. Data sharing is not applicable to this article.
